# Seasonal dimorphism in the horny bills of sparrows

**DOI:** 10.1002/ece3.474

**Published:** 2013-01-11

**Authors:** Russell Greenberg, Matthew Etterson, Raymond M Danner

**Affiliations:** 1 Smithsonian Migratory Bird Center, Smithsonian Conservation Biology InstituteNational Zoological Park, Washington, District of Columbia, 20008; 2 Office of Research and Development, National Health and Environmental Effects Research Laboratory, Mid-Continent Ecology Division, U.S. Environmental Protection AgencyDuluth, Minnesota, 55804

**Keywords:** Bill size, bird beaks, emberizidae, rhamphotheca, salt marsh birds, sexual selection, tidal marsh birds

## Abstract

Bill size is often viewed as a species-specific adaptation for feeding, but it sometimes varies between sexes, suggesting that sexual selection or intersexual competition may also be important. Hypotheses to explain sexual dimorphism in avian bill size include divergence in feeding niche or thermoregulatory demands, intrasexual selection based on increased competition among males, or female preference. Birds also show seasonal changes in bill size due to shifts in the balance between growth rate and wear, which may be due to diet or endogenous rhythms in growth. Insight into the function of dimorphism can be gained using the novel approach of digital x-ray imaging of museum skins to examine the degree to which the skeletal core or the rhamphotheca contribute to overall dimorphism. The rhamphotheca is ever-growing and ever-wearing, varying in size throughout life; whereas the skeletal core shows determinant growth. Because tidal marsh sparrows are more dimorphic in bill size than related taxa, we selected two marsh taxa to investigate dimorphism and seasonality in the size of the overall bill, the skeletal core, and the rhamphotheca. Bill size varied by sex and season, with males having larger bills than females, and bill size increasing from nonbreeding to breeding season more in males. Skeletal bill size varied with season, but not sex. The rhamphotheca varied primarily with sex; males had a larger rhamphotheca (corrected for skeletal bill size), which showed a greater seasonal increase than females. The rhamphotheca, rather than the skeletal bill, was responsible for sexual dimorphism in overall bill size, which was particularly well developed in the breeding season. The size of the rhamphotheca may be a condition-based character that is shaped by sexual selection. These results are consistent with the evidence that bill size is influenced by sexual selection as well as trophic ecology.

## Introduction

The beaks of birds have received considerable attention, primarily as a morphological adaptation shaped by the varying trophic ecologies of species (Darwin 1859; Grant 1986). An increasing number of studies suggest that other factors, such as sexual selection (Price 1984; Babbitt and Frederick 2007; Chaine and Lyon 2008) and thermoregulation (Symonds and Tattersall 2010), may influence this primarily trophic structure. The avian bill, however, is not a fixed feature for any species, and any analysis of function should account for variation with age, sex, and time of year. Sexual dimorphism in bill size and shape is of particular interest because it may reflect selection based on divergent trophic ecology between sexes, divergent sex roles, or intrasexual competition. Documented cases of pronounced sexual dimorphism are found in a number of taxonomic groups, geographic regions, and foraging guilds. Although it appears that such dimorphism is often found in island species (Selander 1966), and may be particularly prevalent in woodpeckers (Selander 1966), hummingbirds (Temeles et al. 2000; Berns and Adams 2010), and shorebirds (Szekely et al. 2000), no systematic survey of the correlates of bill size dimorphism has been published, and the proximate and ultimate causes remain poorly understood. Sexual bill dimorphism is far more frequent in Emberizid sparrows of coastal salt marshes than in related species or subspecies (Rising 1987; Greenberg and Olsen 2010) from other habitats, making this ecosystem an excellent focus for the adaptive significance of bill dimorphism.

The prevailing hypothesis for bill dimorphism is the reduction in intersexual competition for food resources (Selander 1966; Temeles et al. 2000; Radford and du Plessis 2004). This is often thought to occur where interspecific competition is decreased and therefore the two sexes can diverge in their foraging niche (Gosler 1987). During the breeding season, this could result in more efficient harvesting of resources to provision young. However, dimorphism in trophic structures (such as dentition or jaw musculature) among nonavian species, including lizards, primates, carnivores, and pseudoscorpians, suggests that intrasexual selection, particularly male–male contest, could play a role as well (Vitt and Cooper 1985; Shine 1989; Gittleman and Van Valkenburgh 1997; Thorén et al. 2005). Success in male–male conflict (with the bill as a weapon) has occasionally been suggested as the selective force underlying bill dimorphism in birds (Babbitt and Frederick 2007; Greenberg and Olsen 2010). Bills also play a role in convecting heat and, hence, thermoregulation (Tattersall et al. 2010). Greenberg et al. (2012) suggested that tidal marsh sparrows have large bills, particularly in hot regions, to convect heat without losing water through evaporation. Male sparrows may be under greater selection to use their bill as a heat radiator because they need to remain exposed and active to maintain their territories. Finally, bill size in males may respond to female choice. Kimball (1996) demonstrated female preference for male house sparrows (*Passer domesticus*) with larger (deeper) bills, and Price (1984) determined that females differentially mated with male medium ground finches (*Geospiza fortis*) with deeper and wider beaks.

Bird bills consist of a keratin sheath (rhamphotheca) surrounding a skeletal core (premaxilla and mandible). The skeletal elements are generally thought to display determinate growth (although the age at which growth ceases is poorly known in most species) and the rhamphotheca grows and wears continuously throughout life (Matthysen 1989). Although the skeletal core sets the basic architecture of the bill and may have an important influence over total size, much of the interindividual variation can be attributed to variation in the rhamphotheca (e.g., see Borras et al. 2002).

Heritability has been found to be relatively high for bill size in birds (Smith and Zach 1979; Boag 1983), which has led to a search for the genetic controls on bill size. While much progress has been made in determining the role of developmental genes in controlling variation in skeletal bill size in birds (Abzhanov et al. 2004, 2006; Badyaev et al. 2008), the factors that control variation in rhamphotheca size, both within and between individuals, has received little study. Some recent work has begun to focus on possible pathologies that might affect abnormal bill growth (Handel et al. 2010; van Hemert et al. 2012a); little is known about proximate control of “normal” rhamphotheca size.

Because the rhamphotheca is ever-growing, studying the factors that contribute to its variation may be critical to understanding plasticity in overall bill size variation. In particular, a large contribution of the rhamphotheca to overall bill size dimorphism could provide a case for examining bill size as a condition-based trait that is important in sexual selection vis-à-vis male interactions and female choice. Greater breeding season investment in rhamphothecal growth for the sex with greater reproductive competition (males in this case, Andersson 1994) would provide some evidence for the role of sexual selection in bill size dimorphism. Differences between the sexes in seasonal variation in growth has been described for the knobs on bills of certain species (Horrocks et al. 2009) and knob size has been shown to affect male–male competition and reproductive success (Ferns et al. 2005), but the functional part of the bill of birds has not been similarly investigated.

In this study, we examine the contribution of sex and time of year to the variation in bill size in two taxa of tidal marsh sparrows that have previously been reported to show bill size dimorphism during the breeding season. We first modeled the effect of sex and date on overall bill size based on a large series of museum study skins. We then examined the variation in skeletal bill size and rhamphotheca using digitally measured, high-resolution radiographs in a subset of these specimens.

## Methods

### Focal Taxa

**Figure 1:**
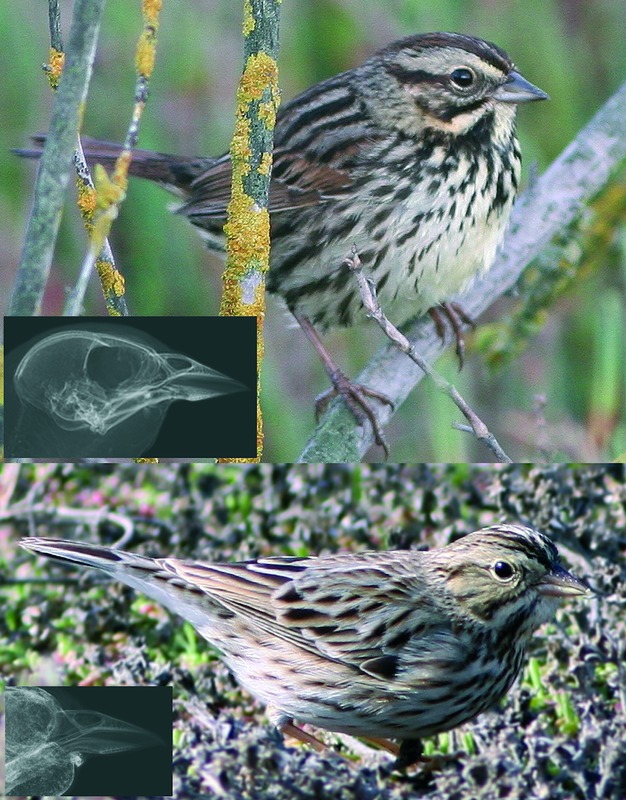
Photographs and radiographs of Alameda song sparrow (top) and Belding's savannah sparrow (bottom). Photographs courtesy of John Sterling.

The study focused on the Belding's savannah sparrow (Belding's sparrow, *Passerculus sandwichensis beldingi*) and the Alameda song sparrow (Alameda sparrow, *Melospiza melodia pusillula*) (Fig. 1). These two taxa were selected because they show sexual bill dimorphism during the breeding season (Greenberg and Olsen 2010), were represented by sufficient museum material, and are permanently resident so that samples taken throughout the year are from a local population. The Belding's sparrow is resident from southern California to southern Baja California (all samples were from southern California marshes) and breeds from March to July (Bent 1968); the Alameda sparrow is resident along the South San Francisco Bay and breeds from late February to June (Johnston 1954).

### Measurements

**Figure 2:**
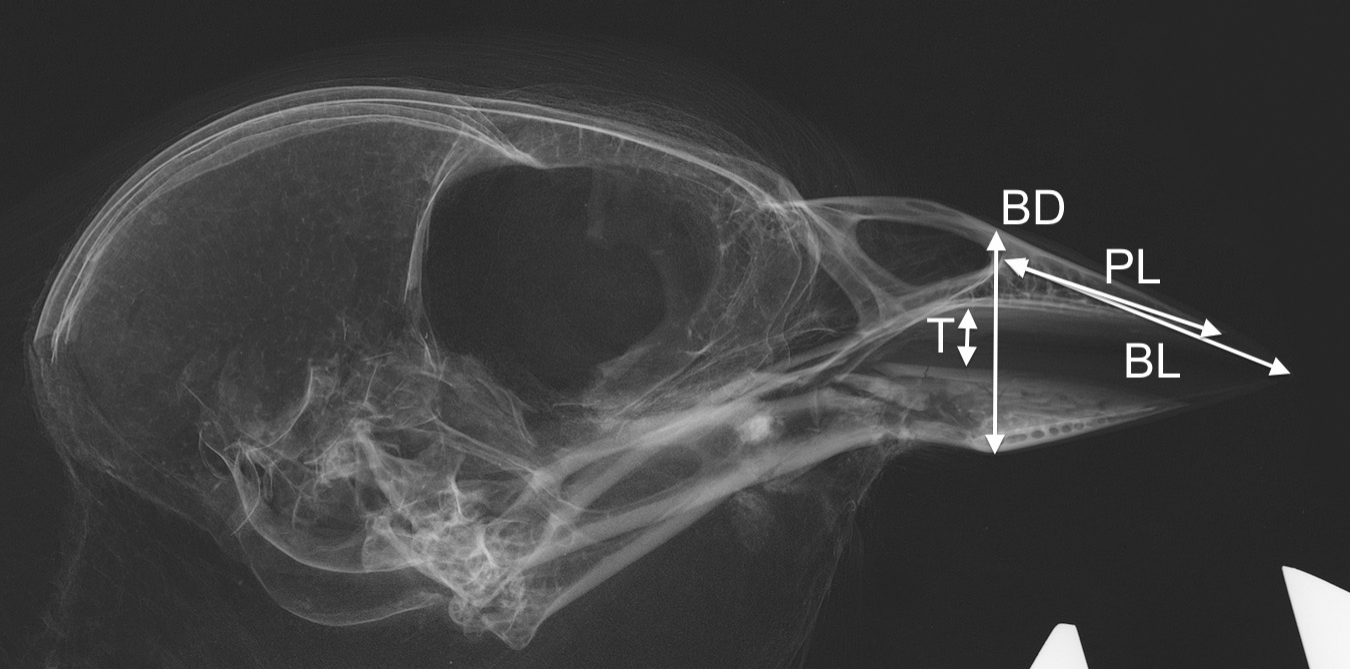
Radiograph of a sparrow showing the measurements taken for calculations of skeletal bill size: BL = Bill Length; PL = Premaxillary Length; BD = Bill Depth; T = Tomium.

The lead author measured study skins of 347 (205♂, 142♀) Alameda and 388 (226♂, 162♀) Belding's sparrows; he did not know the date or sex of the specimen at the time of measurement. The specimens were collected primarily in the first decades of the 20th Century and are, on average, approximately 90 years old. We excluded all specimens in juvenile plumage. Measurements included bill length, depth, and width at the anterior edge of the nares. Collection date and location, along with sex, were available on the specimen tag. We took digital radiographs of the head of a subsample of 200 museum study skins of the Alameda sparrow (119 ♂, 81♀) and 132 specimens of the Belding's sparrow (72♂, 60♀) using a Kevex Microfocus x-ray source (PXS10-16W, Thermo Scientific - Kevex X-Ray Products, Scotts Valley, CA) with a 6 micron focal spot along with a Varian System Flat Panel Amorphous Silicon Digital X-Ray Detector (PaxScan 4030R, Varian Medical Systems, Inc., Palo Alto, CA) with a 28.2 × 40.6 cm pixel area. This produced a 7.1 MB 8 bit TIFF file, which was captured by Viva k.03 software (Varian Medical Systems, Inc.). The image was transferred to ImageJ (Rasband 2012) where the lead author measured both the bony (pre- and inferior maxillary bones) and rhamphothecal (keratin) elements of the bill to determine which tissue type contributed to any differences in bill size. In these images, skeletal elements are visible within the outline of the rhamphotheca. The sex or collection date for the specimen was not on the image when the measurements were taken. The measurements used in this study are bill depth (BD) at the anterior edge of the nasal cavity, tomium depth (T, distance between premaxillary and inferior maxillary bones at the same location as the BD measurement), premaxillary (PL) and bill (BL) lengths from the anterior edge of the nasal cavity, and bill tip (BT), the difference between PL and BL. A sample skull showing four sample measurements is depicted in Fig. 2. An estimate of surface area of the cone of the skeletal bill was obtained as follows: (BD-T)/2*PL*π, substituting (BD-T)/2 for radius because only a lateral image was taken. This results in a slight overestimation as BW is slightly less than BD. In addition, there is a gap at the distal end of the maxillae causing a slight overestimation, but these measurement errors are unlikely to have biased the results. Total bill surface area was the same except we substituted BL for PL and BD for BD-T.

### Analysis

We used linear models (GLM module of Statistica version 10, StatSoft, Inc. 2011) to predict total bill size (surface area) as a function of sex (coded as a dummy variable with female as the reference category) and collection date. We tested five models: intercept only (Null), sex, date, sex + date, and sex + date + sex * date. Diagnostics based on residual plots were conducted on the top models to insure that they conformed to the assumptions of normally distributed random errors, linearity, and homoscedasticity (for this and the radiographic data below). For the radiographic measurements, the residuals from the ordinary least squares regression between skeletal bill surface area and total bill surface area (as estimated from radiograph) were used as an estimate of rhamphotheca size independent of underlying skeletal size. We also modeled skeletal bill surface area. Sex, date, and the interaction between these were used as potential predictor variables. We compared the performance of alternative models using AIC_*c*_ (Anderson 2008) and the evidence ratio (the ratio of the model probabilities [*w*]). The effect of individual parameters was compared using the standardized partial regression coefficient (β) from the global model. Predicted values were derived by model averaging when more than one model had a ΔAIC_*c*_ value of less than 2 and, in those cases unconditional standard errors are presented (Anderson 2008).

Collection date was first assigned a number between 1 and 365 with the year beginning on August 16. This date was selected arbitrarily from a time period when hatching year birds that have completed post juvenile molt begin appearing in the collections. Since date is modeled (as follows) as a cyclical function, the starting date does not affect the overall model fit, but only the relative contribution of the two combined trigonometric functions. To model date as a cyclical function, collection date was then twice transformed. First, we created the variable dt in radians using the formula: 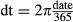 then dt was transformed to *cos* (dt) and *sin* (dt). The two terms *cdt* and *sdt* together creates an oscillating response that forces the predicted value for bill size to be equivalent at date = 0 and date = 365 (Zar 1999). Because sdt accounted for substantial amounts of variation in nearly all models, but cdt explained almost no variation and was not significant in any model, we dropped the cdt term from the analyses to economize on parameters used to describe date.

## Results

### Overall bill size variation

**Figure 3:**
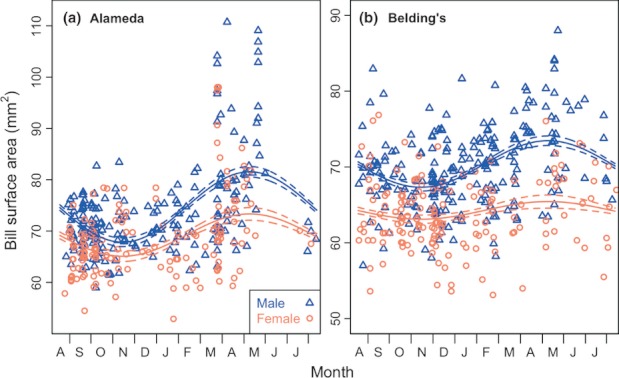
Bill surface area plotted by collection date (1–365 starting at August 16) and separated by sex for A) Alameda song sparrow and B) Belding's savannah sparrow. Lines represent predicted values ± standard errors.

The best supported models for total bill size in both the Alameda and Belding's sparrow included an interaction between sex and date (Fig. 3, Table 1). For the Alameda sparrow, the interaction model with sex and date had a model weight of 0.76 and was 1.94 × 10^23^ more likely than the null model. The model with both sex and date and their interaction was also the top model for the for Belding's sparrow (*w* = 0.859), which was 2.00 × 10^33^ times more likely than the null model. These models show that males have larger bills than females, both sexes have larger bills during the breeding season, and there was a distinctly greater difference between the sexes during the late breeding season (April–July) in both species (Table 2, Fig. 3), because male bill size increased more than females as breeding approached.

**Table 1:** Parameters, AIC_*c*_, ΔAIC_*c*_, and model weights for linear models relating sex and collecting date to the total bill surface area in museum specimens of Alameda and Belding's sparrows

		Alameda sparrow			Belding's sparrow		
			
Model	K	AIC_*c*_	ΔAIC_*c*_	*w*	AIC_*c*_	ΔAIC_*c*_	*w*
sdt + sex + sdt * sex	4	1437.12	0.00	0.760	1239.81	0.00	0.859
sdt + sex	3	1439.42	2.30	0.287	1243.60	3.62	0.141
sdt	2	1471.30	34.19	2.87 × 10^−8^	1282.37	42.39	5.36 × 10^−10^
sex	2	1520.42	83.30	6.20 × 10^−19^	1349.18	109.20	1.67 × 10^−24^
Null	1	1544.36	107.25	3.91 × 10^−24^	1393.94	153.36	4.29 × 10^−34^

**Table 2:** Standardized regression coefficients (β) and their 95% confidence intervals for linear models relating sex, sine of date, and their interaction with surface areas of the total bill, rhamphotheca, and skeletal bill in Alameda and Belding's sparrows. For sex, female was the reference category

Taxon	Measurement	*N*	sex (male)	sdt	sex * sdt
Alameda sparrow	Total Bill	366	0.30 (0.21 to 0.39)	−0.54 (−0.65 to −0.42)	0.14 (0.02 to 0.26)
	Rhamphotheca	200	0.49 (0.37 to 0.62)	−0.35 (−0.51 to −0.19)	0.25 (0.08 to 0.41)
	Skeletal Bill	200	0.08 (−0.05 to 0.22)	−0.37 (−0.54 to −0.20)	−0.06 (−0.24 to 0.11)
Belding's sparrow	Total Bill	388	0.51 (0.40 to 0.62)	−0.58 (−0.83 to −0.33)	0.33 (0.08 to 0.59)
	Rhamphotheca	132	0.45 (0.25 to 0.65)	−0.65 (−1.14 to −0.16)	0.52 (0.01 to 1.03)
	Skeletal Bill	132	0.01 (−0.20 to 0.18)	−0.48 (−0.95 to −0.01)	0.00 (−0.49 to 0.49)

### Sexual dimorphism in bill size components

The Alameda sparrow was significantly dimorphic in tomium depth, bill tip length, and premaxillar length (Table 3). Percent dimorphism was greater in the rhamphothecal elements (10–14%) than the skeletal measurements (−1 to 3%). Similarly, skeletal measurements were not significantly sexually dimorphic in the Belding's sparrow (−1 to 2%, Table 4), but the rhamphothecal measurements were significantly dimorphic or nearly so (8–10%).

**Table 3:** Mean (standard deviation) and percent dimorphism of bill measurements (mm) for the Alameda sparrow (117♂, 81♀). Bolded *P* values indicate significance with an alpha = 0.0125, with a Bonferroni correction for a desired alpha = 0.05

Feature	♂	♀	% dimorphism (95% CI)	*t*-value	*P*
Premaxillar Length	6.87 (0.34)	6.69 (0.32)	2.75 (1.70 to 3.70)	3.76	**0.000**
Skeletal Bill Depth	4.34 (0.26)	4.37 (0.30)	−0.79 (−2.07 to 0.49)	−0.85	0.396
Bill Tip Length	1.92 (0.26)	1.69 (0.26)	13.52 (10.09 to 16.95)	6.03	**0.000**
Tomium Depth	1.80 (0.19)	1.63 (0.19)	10.19 (7.73 to 12.65)	6.00	**0.000**

**Table 4:** Mean (standard deviation) and percent dimorphism of bill measurements (mm) for the Belding's sparrow (72♂, 60♀). Bolded *P* values indicate significance with an alpha = 0.0125, with a Bonferroni correction for a desired alpha = 0.05

Feature	♂	♀	% dimorphism (95% CI)	*t*-value	*P*
Premaxillar Length	7.40 (0.57)	7.29 (0.45)	1.56 (−0.17 to 3.29)	−1.26	0.211
Bony Bill Depth	4.34 (0.36)	4.40 (0.35)	−1.25 (−3.32 to 0.55)	0.89	0.374
Bill Tip Length	2.07 (0.39)	1.92 (0.34)	7.82 (3.29 to 12.35)	−2.32	0.022
Tomium Depth	1.85 (0.34)	1.68 (0.23)	9.83 (5.73 to 14.47)	−3.16	**0.002**

### Determinants of rhamphothecal variation

**Figure 4:**
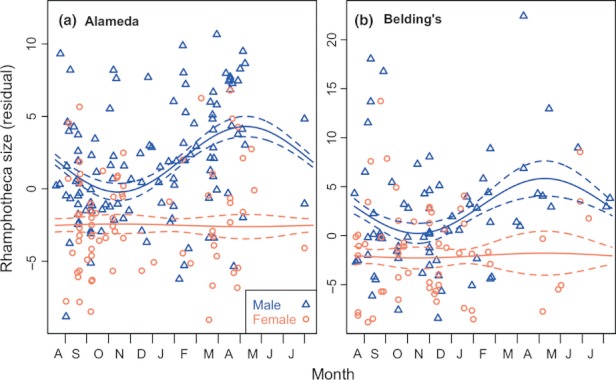
Rhamphotheca size (residual value) of male and female A) Alameda song sparrows and B) Belding's savannah sparrows throughout the year. Lines represent (model average) predictions ± (unconditional) standard errors.

The date and sex interaction models for rhamphotheca size received the greatest support in both the Alameda sparrows (*w* = 0.944, Table 5) and the Belding's sparrows (*w* = 0.608, Table 5) and the best model (interaction) was 7.10 × 10^11^ and 6.08 × 10^3^ times more likely than the null model in the two taxa, respectively. The model that included the interaction term received more support than the simple additive model (evidence ratios = 17.48 and 1.98 for Alameda and Belding's sparrow, respectively). The absolute value for βs (effect size) for Alameda song sparrow was largest for sex (0.49) followed by sdt and sex * sdt. None of the confidence intervals intersected the 0 line (Table 2). For the Belding's sparrow, β was largest for sdt (0.65) and large for sex and sdt as well and none of the confidence intervals intersected the 0 line. The highest ranked model and its βs show that males have a relatively large rhamphotheca (corrected for skeletal bill size) and that rhamphothecal size increases from nonbreeding to breeding season, particularly in males (Table 5, Fig. 4).

**Table 5:** Parameters, AIC_*c*_, ΔAIC_*c*_, and model weights for linear models relating sex and collecting date to the size of the rhamphotheca (the residual of skeletal bill vs. total bill regression) based on radiographs of museum specimens of Alameda and Belding's sparrows

		Alameda sparrow			Belding's sparrow		
			
Model	K	AIC_*c*_	ΔAIC_*c*_	*w*	AIC_*c*_	ΔAIC_*c*_	*w*
sdt + sex + sdt * sex	4	564.79	0.00	0.944	489.14	0.00	0.608
sdt + sex	3	575.14	5.73	0.054	490.51	1.37	0.306
sex	2	581.71	12.30	2.02 × 10^−3^	493.09	3.94	0.085
sdt	2	618.08	48.67	2.55 × 10^−11^	501.64	12.50	0.001
Null	1	624.012	54.58	1.33 × 10^−12^	506.142	17.00	0.0001

**Table 6:** AIC analysis for models that relate skeletal bill size to sex and date in Alameda and Belding's sparrows

		Alameda sparrow			Belding's sparrow		
			
Model	k	AIC_*c*_	ΔAIC_*c*_	*w*	AIC_*c*_	ΔAIC_*c*_	*w*
sdt	2	555.19	0.00	0.587	509.24	0.00	0.689
sex + sdt	3	556.14	0.95	0.347	511.19	1.94	0.261
sex * sdt	4	558.75	3.56	0.094	514.49	5.24	0.050
sex	2	588.73	33.54	2.91 × 10^−8^	545.67	36.43	8.47 × 10^−9^
Null	1	589.82	34.63	1.69 × 10^−9^	538.97	29.75	2.42 × 10^−7^

### Determinants of skeletal bill size variation

**Figure 5:**
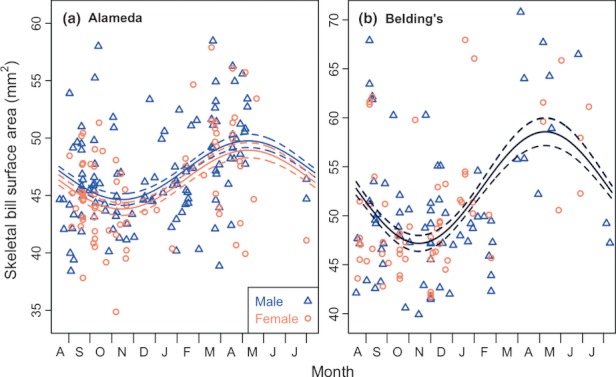
Surface area of the skeletal bill of male and female A) Alameda song sparrows and B) Belding's savannah sparrows throughout the year. Lines represent model average predictions ± unconditional standard errors.

The date-only models received the greatest support for skeletal bill size in both the Alameda sparrow and the Belding's sparrow (Table 6). The model weight for date was 0.587 and 0.689 for the two taxa, respectively. These models were 3.47 × 10^8^ and 2.85 × 10^6^ times more likely than the null model, respectively. Within the global model (sex, date and interaction), only sdt had large βs among the independent variables for skeletal bill size (Table 2), −0.37 and −0.48 for Alameda sparrow and Belding's sparrow, respectively. The model ranking and β value suggest a seasonal variation in average skeletal bill size in the absence of any sex-related differences (Fig. 5).

## Discussion

Both taxa showed both seasonal variation and sexual dimorphism in overall bill size. Males had larger bills than females and bill size increased from the nonbreeding to the breeding season with the nadir occurring in the early winter and the apex in late spring to mid-summer (the end of the breeding season for both taxa). Both taxa showed greater sexual dimorphism in the rhamphothecal than the skeletal measurements of bill size. Furthermore, in the global models of both species the standardized effect size (β) was large (0.49, 0.45) for sex. In contrast, sex was an unimportant and date an important variable for explaining skeletal bill size. Support was provided for the importance of an interaction between sex and date for both overall bill size and rhamphothecal size, suggesting that the male rhamphotheca increases in size at a faster rate than the female rhamphotheca prior to the breeding season.

The causes for sexual dimorphism and seasonal variation in the rhamphotheca can be addressed at both a proximate and ultimate level. The proximate basis for variation in bill size can result from differences in growth rate or in bill wear. Growth rate could vary as a result of endogenous rhythms controlled by photoperiod or hormonal state, or the nutritional status of the bird. van Hemert et al. (2012b) found that bill growth rate decreased in black-capped chickadees (*Poecile atricapillus*) while in captivity from December through April, although it remains unclear if this decline in growth was part of a naturally occurring annul cycle or a result of captivity. Although the effect of nutrition and photoperiodicity specifically on rhamphothecal growth is generally not known, support for these mechanisms can be found for other keratin-based structures. The effect of ambient temperature on nutritional status has been demonstrated in affecting the growth rate of horns in sheep (Hoefs and Nowlan 1997; Giacometti et al. 2002) and photoperiod has been implicated in breeding season increases in horn growth as well (Santiago-Moreno et al. 2012). Seasonal variation in growth rate has been documented in bovine hoofs, another keratinized structure (Hahn et al. 1986).

Sexual dimorphism in rhamphothecal size could, as has been shown in the horns of ungulates, result from differential investment of proteins and other nutrients, into the growth of the keratin structure. Although not related specifically to sex or individual differences, the idea of bill growth being influenced by competing allocations was suggested by Morton and Morton (1987) in their study of white-crowned sparrows (*Zonotrichia leucophrys*). They provided evidence that the onset of bill growth in the spring corresponds to a switch to an insect diet, but that the decline in bill size in the fall may be due to allocation of resources to prebasic molt.

On the wear side of the equation, bill size is known to vary due to variation in the rate of wear resulting from differences in diet or feeding intensity (Matthysen 1989). This is the most popular explanation for seasonal variation in bill size in birds (Hulscher 1984). So, for example, the decrease in bill size found in finches and sparrows during the autumn and winter may be related to the shift from insectivory to granivory (Clancey 1948; Davis 1954, 1961; Johnson 1977). This might be an adequate explanation for seasonal changes in bill size in these taxa. However, diet data from salt marsh song sparrows suggests that they depend much less on seeds and are more insectivorous than other song sparrows (Aldrich 1984; Grenier 2004). Few data on nonbreeding diet of tidal marsh sparrows exist, but the abundance of seeds in salt marshes is thought to be very low compared to habitats occupied by nontidal marsh populations of song and savannah sparrows (Leck 1989). With regards to the sex difference, unless the sexes differ substantially in their diet throughout the year (and particularly during the breeding season), differential bill wear is not a viable explanation for the sexual dimorphism in the rhamphotheca. At this point, there is no evidence from the natural history of these subspecies to suggest that such a difference exists.

If the difference in sex is not simply the result of differential wear, then adaptive (ultimate) explanations need to be considered. The possible ultimate explanations for bill-size dimorphism, in general, center on divergence in foraging niche or sexual selection. With respect to the trophic divergence hypothesis, little evidence supports the existence of an intrinsic difference in skeletal bill size. Such a difference in skeletal bill size would indicate variation in the potential speed or force associated with the foraging apparatus, in turn associated with a significant divergence in diet or foraging mode between the sexes. The restriction of dimorphism to the rhamphotheca is more consistent with males investing more nutritional resources into rhamphothecal growth for reasons other than foraging, particularly during the breeding season.

Males could invest more in rhamphothecal growth for several different reasons. First, because growth is concentrated in the bill tip and the cutting edges (tomia) of the bill, increases in the size of these two features might be related to the use of the bill as a weapon in male–male interactions. Greenberg and Olsen (2010) hypothesized that increased bill size dimorphism in tidal marsh sparrows might be related to high breeding densities and the potential for increased male–male interaction in this habitat. Second, large bills may signify an ability of males to sequester resources and thus be an honest signal of male quality for either male–male interactions or female choice. A range of other structures, such as the ornamental feathers of birds and the horns of beetles, have recently been demonstrated to grow differentially large because of increased sensitivity to insulin or insulin-like growth factors, which provides the basis for growth being an honest signal of nutritional condition (Emlen et al. 2012). Third, enhanced investment might increase heat loss to the surrounding air to aid in thermoregulation (Tattersall et al. 2010). Increased rhamphothecal growth is associated with a greater blood supply in the basal layers beneath the cornified outer layer, and bill surface area is tightly correlated with high summer temperatures across all North American tidal marsh endemics (Greenberg et al. 2012). Thermoregulation might be a particularly acute issue for territorial males, patrolling their territories and singing from exposed perches (Greenberg et al. 2012). This hypothesis would therefore explain an increase during summer in size in both sexes with a greater increase in males. Furthermore, maximum bill size is not achieved until late in the breeding season (Fig. 3), well after territorial acquisition and mate selection occur, but before the period of maximum temperatures, which supports the thermoregulation hypothesis.

The reason for a seasonal increase in skeletal bill size remains unclear. Age could be a factor. As a substantial majority of the specimens were probably first year birds, the growth of young birds could contribute to a seasonal change in average bill size. Price and Grant (1984) and Smith et al. (1986) found that Darwin's Finches and song sparrows show increased bill size throughout the first year. This could explain the pattern of increased overall bill size. Skeletal growth is generally considered to be determinate and adult bill size is achieved early in life. As far as we know, however, it has not been shown that skeletal bill size continues to increase after the first months of development in songbirds.

Another possible explanation lies with the nature of the sample. The museum study skins provide a cross section of bill size collected throughout the year over many years. Changes in the average bill size of these samples may reflect changes in the population due to the effects of selection over the course of the year (Johnston and Fleischer 1981). Additionally, specimens collected during the breeding season are probably biased towards those birds that successfully acquired a territory. Only longitudinal radiographic studies of individuals will establish if an extended period of skeletal growth, selection, or a sample biased towards territory holders underlies the seasonal increase in skeletal bill size.

## Conclusions

This study establishes that significant seasonal and sex-based variation can be found in the bill size of two subspecies of tidal marsh sparrows. Bill size is smallest in the late autumn and increases to its maximum in the late spring/early summer (late in the breeding season for these species). Males tend to have larger bills than females. More importantly, the use of high-resolution x-ray allowed us to partition this variation into skeletal and keratin elements. Mean skeletal bill size varied by date and mean rhamphothecal size varied primarily by date and sex, with males having a larger rhamphotheca even when corrected for variation in skeletal bill size. There is good support for the differential increase in total bill and rhamphotheca size in males during the breeding season. The most likely explanation for the increase in rhamphothecal size in males is that males invest more in its growth than females, particularly in the breeding season. This finding opens the possibility that sexual selection influences male bill size, with the rhamphotheca serving as a condition-based character. It also provides further impetus for more research to determine the proximate factors that influence rhamphothecal size and shape.
